# Machine learning-based early warning system for hemodynamic deterioration in cardiovascular ICU patients: a bidirectional cross-validation study

**DOI:** 10.3389/fcvm.2025.1694001

**Published:** 2026-01-21

**Authors:** Shicheng Gao, Yunhai Zhang, Menghua Deng, Haohui Liu, Weixian Xu, Meng Luo, Ying Tian, Bin Zhang

**Affiliations:** Critical Care Department, The Eighth Clinical Medical College of Guangzhou University of Chinese Medicine, Foshan, China

**Keywords:** cardiovascular ICU, critical care, early warning systems, external validation, hemodynamic monitoring, machine learning

## Abstract

**Background:**

Early identification of hemodynamic deterioration in cardiovascular intensive care unit (ICU) patients is critical for improving clinical outcomes. Traditional monitoring approaches and scoring systems often fail to capture dynamic multidimensional physiological changes, and existing machine learning models frequently lack robust external validation across diverse healthcare systems.

**Methods:**

We employed a retrospective multi-center cohort design to develop machine learning prediction models using the MIMIC-IV database (46,007 admissions) and the eICU database (50,949 admissions). To rigorously assess model robustness and generalizability, a novel bidirectional cross-validation framework was implemented: models were trained on MIMIC data and validated on eICU data, and conversely, trained on eICU data and validated on MIMIC data. The study defined a strict composite outcome comprising hemodynamic instability, tissue hypoperfusion, and confirmed cardiac etiology. Multiple machine learning algorithms were evaluated to identify the optimal classifier.

**Results:**

The Random Forest model was selected as the optimal classifier. Bidirectional validation demonstrated exceptional cross-database generalizability: the MIMIC-trained model achieved an Area Under the Receiver Operating Characteristic (AUROC) of 0.841 on the eICU cohort, while the eICU-trained model achieved an AUROC of 0.852 on the MIMIC cohort, with performance degradation controlled within a minimal range (<4%). DeLong tests confirmed that the model significantly outperformed traditional clinical scores, including SOFA (AUROC 0.681) and APACHE II (AUROC 0.747). The five-level risk stratification system exhibited a strict monotonic increase in mortality rates, ranging from 0.8% in the very low-risk group to 84.2% in the very high-risk group. SHAP analysis identified hemoglobin, history of acute myocardial infarction, and creatinine as the most significant predictors.

**Conclusions:**

We successfully developed and validated a machine learning-based early warning system for hemodynamic deterioration in cardiovascular ICU patients. The bidirectional cross-validation approach provides robust evidence for model generalizability, while the multi-level risk stratification system and SHAP-based interpretability offer practical clinical decision support. This system demonstrates significant potential to enhance early identification rates, improve patient outcomes, and optimize healthcare resource utilization efficiency.

## Introduction

Hemodynamic deterioration in cardiovascular intensive care unit (ICU) patients represents one of the most critical and time-sensitive challenges in modern critical care medicine. Early identification of patients at risk for hemodynamic instability can substantially improve clinical outcomes, reduce mortality, and optimize resource utilization (1, 2). However, despite decades of technological advancement, traditional monitoring approaches remain constrained (3, 4). Conventional approaches, including pulmonary artery catheterization, central venous pressure monitoring, and arterial blood pressure measurement, provide valuable but incomplete information about cardiovascular status (5–7). These methods frequently lack the sensitivity to detect early physiological compromise, failing to predict clinical deterioration until substantial, and potentially irreversible, derangement has occurred (8–12).

To augment clinical judgment, scoring systems such as APACHE II and SOFA are widely used for risk stratification. While these tools demonstrate robust performance in predicting overall mortality—with reported sensitivities exceeding 89% in general cohorts—they were not designed for the dynamic, real-time prediction of hemodynamic instability (13–18). While APACHE II shows sensitivity of 89.9% and specificity of 97.6%, and SOFA demonstrates 90.1% sensitivity and 96.6% specificity in mortality prediction, these systems were not specifically designed for hemodynamic deterioration prediction and may lack the temporal resolution and feature complexity needed for early warning applications (19–21). Furthermore, these scoring systems typically provide static assessments rather than continuous monitoring capabilities essential for real-time clinical decision-making.

The integration of machine learning (ML) technologies into critical care has emerged as a promising solution to address these limitations (22–24). Recent advances in artificial intelligence have demonstrated significant potential for improving prediction accuracy in intensive care settings through sophisticated analysis of electronic health record data (25–27). Machine learning-based early warning systems have shown superior performance compared to traditional aggregate-weighted early warning systems, with the ability to incorporate temporal trends and capture complex relationships among physiological parameters that conventional models cannot detect (28, 29).

Several recent studies have specifically focused on cardiovascular and hemodynamic prediction in ICU settings, demonstrating promising results. A systematic review identified 29 primary studies employing 15 different ML techniques for clinical deterioration prediction, highlighting the rapid growth and diversity in this field (30). Early warning systems for circulatory failure have achieved 90% sensitivity with 82% of events identified more than 2 h in advance, demonstrating the feasibility of ML-based early prediction systems (31). Explainable machine learning frameworks for cardiac arrest prediction have demonstrated stable performance across different ICU environments using ensemble learning approaches (32). Multimodal approaches combining demographic, clinical, and vital sign data have been implemented to predict in-hospital cardiac arrest up to 13 h in advance, achieving areas under the ROC curve exceeding 0.85 (33).

The implementation of machine learning early warning systems has shown promising clinical results in real-world settings. A multicenter clinical intervention trial demonstrated that ML-based early warning score implementation was associated with reduced in-hospital mortality, likely driven by earlier and more frequent ICU transfers (34). Clinical evaluation of ML-based early warning systems in general internal medicine units has been associated with lower risk of non-palliative death compared to pre-intervention periods (35). Real-time machine learning models using heart rate variability have achieved areas under the ROC curve of 0.881 for cardiac arrest prediction (36).

Despite these advances, several critical limitations remain in current ML-based prediction systems. First, most existing models rely on single-database development with limited external validation, raising concerns about generalizability across different healthcare systems and patient populations (37, 38). Second, many studies focus on single prediction endpoints rather than the complex, multi-stage nature of hemodynamic deterioration. Third, there is insufficient emphasis on clinical interpretability and actionable risk stratification that can guide real-time clinical decision-making (39, 40).

The concept of bidirectional validation, while increasingly recognized in ML applications, remains underutilized in critical care prediction models (41, 42). This approach, involving mutual validation between independent datasets, can provide more robust assessment of model generalizability and identify dataset-specific biases that may limit clinical applicability. Recent work in healthcare ML has emphasized the importance of external validation and cross-dataset evaluation to ensure real-world deployment readiness, with studies showing that many models exhibit substantial performance degradation when applied to external cohorts (43, 44).

To address these limitations, this study developed a comprehensive machine learning-based early warning system for hemodynamic deterioration in cardiovascular ICU patients using a novel bidirectional cross-validation approach. Our system employs a dual-target variable framework to meet different clinical scenarios: a high-sensitivity screening model for early risk identification and a high-specificity confirmation model for treatment decision support. By leveraging both MIMIC-IV and eICU databases for bidirectional validation, we aim to enhance model robustness and clinical generalizability while providing interpretable, actionable predictions for cardiovascular ICU management.

The primary objectives of this study were to: (1) develop and validate machine learning models for early prediction of hemodynamic deterioration in cardiovascular ICU patients; (2) implement a bidirectional cross-validation strategy to assess model generalizability across different healthcare systems; (3) create a multi-level risk stratification system for personalized clinical decision support; and (4) provide interpretable model predictions through advanced feature analysis to enhance clinical acceptance and adoption.

## Methods

### Study design and data sources

This study employed a retrospective multi-center cohort design to develop machine learning prediction models for early warning of hemodynamic deterioration in cardiovascular ICU patients based on two large critical care databases. Data were sourced from the MIMIC-IV database v3.0 (2008–2019) and the eICU Collaborative Research Database v2.0 (2014–2015), To rigorously assess model robustness and generalizability across different healthcare systems, we implemented a bidirectional cross-validation framework. In this approach, each database served alternately as the derivation cohort for model training and as the independent external validation cohort for testing. Both datasets contain de-identified health information, and data access was granted following the completion of required ethics training and data use agreements.

### Patient selection process and cohort construction

This study employed a systematic patient selection process to establish high-quality cardiovascular ICU cohorts from two large critical care databases. The selection criteria were designed to ensure clinical relevance and data completeness for machine learning modeling. Inclusion criteria comprised adult patients aged 18 years or older who were admitted to cardiovascular ICUs or had cardiovascular disease diagnoses, with an ICU length of stay of at least 24 h and complete baseline clinical data available. Exclusion criteria included patients younger than 18 years, those with ICU stays shorter than 24 h, patients missing more than 50% of key vital signs or laboratory data, and previous heart transplant recipients.

**Figure 1:**
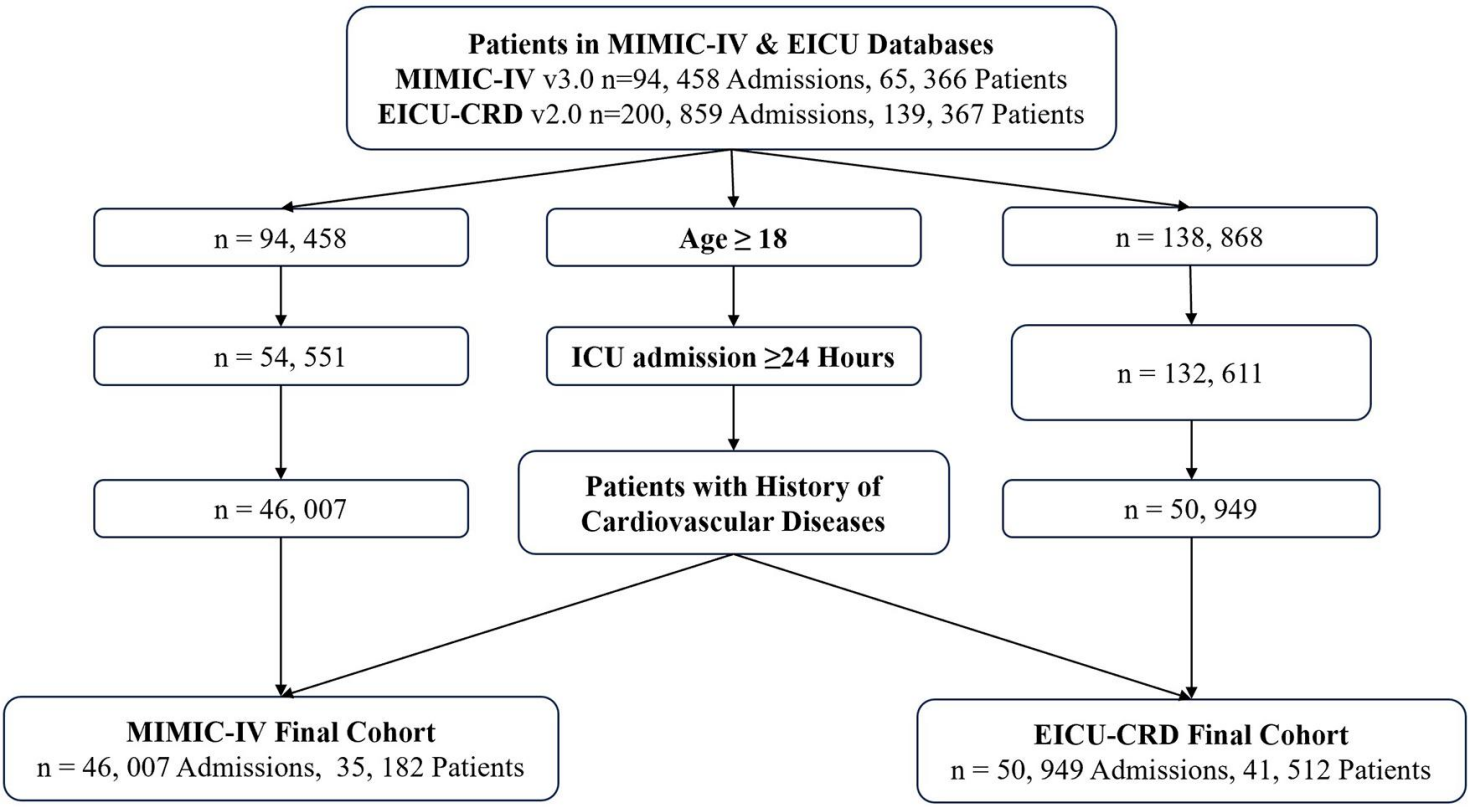
Flowchart.

The sequential screening process is illustrated in Figure 1. Initially, 94,458 admissions from the MIMIC-IV database and 200,859 admissions from the eICU-CRD database were identified. The first screening step excluded pediatric patients (age < 18), reducing the eICU cohort to 138,868 admissions, while the MIMIC-IV cohort remained at 94,458. Subsequently, patients with an ICU length of stay shorter than 24 h were excluded to ensure sufficient observation windows, resulting in 54,551 admissions in MIMIC-IV and 132,611 in eICU-CRD. The final filtering step involved selecting patients with a history of cardiovascular diseases while excluding heart transplant recipients. This rigorous process ultimately yielded a final MIMIC-IV cohort of 46,007 admissions (representing 35,182 unique patients) and a final eICU-CRD cohort of 50,949 admissions (representing 41,512 unique patients). The combined dataset provided a robust foundation for analysis, encompassing a total of 96,956 admissions across both centers.

### Feature selection and data preprocessing

To ensure clinical applicability and strictly prevent data leakage, this study established a rigorous feature auditing process. We first identified and excluded features potentially containing future information through systematic clinical relevance assessment and time-series analysis. 14 safe features were finally included, encompassing demographic characteristics (age, sex), admission timing features, medical history (acute myocardial infarction, heart failure, coronary artery disease, arrhythmia), vital signs (heart rate, mean arterial pressure, respiratory rate, oxygen saturation, temperature), and basic laboratory parameters (hemoglobin, platelets, white blood cells, glucose). All features were clinical indicators available at admission, ensuring model operability in real clinical environments.

Data preprocessing adopted multi-level quality control strategies. To address missing data while preserving the statistical distribution and relationships between clinical features, we utilized the Multivariate Imputation by Chained Equations (MICE) algorithm. This method iteratively imputes missing values by modeling each feature with missing data as a function of other characteristics in the dataset. Outlier detection employed dual standards based on clinical ranges and statistical methods, with values exceeding physiologically reasonable ranges being flagged and manually reviewed.

### Target variable definition and prediction time window

This study establish a rigorous composite outcome, labeled as target_strict to identify high-specificity hemodynamic deterioration defined as simultaneously meeting: hemodynamic instability (1) hemodynamic instability systolic BP < 90 mmHg or MAP < 65 mmHg or Shock Index ≥ 0.9 or early vasopressor use), (2) tissue hypoperfusion (lactate > 2.0 mmol/L or elevated creatinine or acidosis), and (3) a confirmed cardiac etiology.

Regarding feature selection, although the Shock Index (SI) is a critical clinical indicator included in the outcome definition, it was excluded from the input feature set to avoid mathematical redundancy and multicollinearity, as its constituent components (heart rate and systolic blood pressure/MAP) were already included. Instead, the model relied on the non-linear interactions between these raw hemodynamic parameters to capture risk.

The **prediction time window** was designed to support early clinical decision-making: the model utilizes clinical data collected within the **first 24 h of ICU admission** to predict the occurrence of hemodynamic deterioration throughout the subsequent ICU stay.

### Data splitting strategy

To ensure rigorous model evaluation and generalizability, this study adopted a dual-validation framework comprising internal cross-validation and external independent validation, rather than a simple random split.

#### Internal validation

The MIMIC-IV dataset served as the development cohort. We employed 10-fold stratified cross-validation to assess model stability and prevent overfitting. In this process, the dataset was randomly partitioned into 10 equal-sized subsamples, ensuring the proportion of the target variable (target_strict) remained consistent across folds. The model was trained on 9 folds and validated on the remaining fold, with the process repeated 10 times.

#### External validation

To evaluate the model's robustness across different healthcare systems, the eICU-CRD dataset was utilized as a strictly held-out external validation set. The model trained on the full MIMIC-IV cohort was directly applied to the eICU cohort without any retraining or parameter tuning. This approach strictly prevents data leakage and simulates a real-world clinical deployment scenario where the model encounters entirely new patient populations.

### Machine learning modeling strategy

To ensure robust clinical applicability, we adopted a two-phase modeling approach.

Phase 1: Algorithm Selection. We evaluated eight distinct machine learning algorithms, ranging from traditional Logistic Regression to advanced ensemble methods like Random Forest and XGBoost. To ensure a fair comparison, all models underwent rigorous hyperparameter tuning and class balancing to handle the uneven distribution of patient outcomes. The best-performing models were selected based on their stability and predictive accuracy across internal validation.

Phase 2: Generalizability and Risk Stratification. To test whether our models work in different hospital settings, we employed a bidirectional external validation strategy: a model trained on MIMIC-IV was tested on eICU-CRD, and vice versa. This rigorous “stress test” ensures the findings are not specific to a single institution.

Finally, to translate predictions into actionable clinical insights, we developed a dynamic risk stratification system. Instead of a simple “yes/no” prediction, patients were categorized into hierarchical risk groups (e.g., Low, Moderate, High, Critical). The classification thresholds were optimized not just mathematically (using the Youden Index), but also clinically, prioritizing the sensitivity to detect high-risk patients early.

### Statistical analysis and model interpretability

Model interpretability was enhanced through SHapley Additive exPlanations (SHAP) analysis, a game-theoretic approach that provides feature-level explanations for individual predictions (45). SHAP values quantify the contribution of each feature to the model's output, enabling clinicians to understand the reasoning behind predictions and identify the most influential clinical variables (46, 47). This interpretability framework is essential for clinical adoption, as healthcare professionals require transparent, explainable models to make informed treatment decisions (48).

## Results

The study included a total of 96,956 patients across two large databases. The MIMIC-IV cohort comprised 46,007 patients, while the eICU-CRD cohort included 50,949 patients.

The MIMIC-IV cohort was slightly older (mean age 67.4 ± 14.6 vs. 64.4 ± 15.8 years, *P* < 0.001) and had a higher proportion of male patients (59.0% vs. 56.8%, *P* < 0.001) compared to the eICU-CRD cohort (Table 1). While vital signs showed statistical differences due to the large sample sizes, clinical values were generally comparable, though patients in MIMIC-IV presented with slightly lower mean arterial pressure (79.0 ± 10.9 vs. 81.2 ± 14.1 mmHg).

**Table 1:** Baseline characteristics.

Characteristic	MIMIC-IV (*n* = 46,007)	eICU-CRD (*n* = 50,949)	*P* value
Demographics			
Age (years)	67.4 ± 14.6	64.4 ± 15.8	<0.001
Male gender, *n* (%)	27,128 (59.0)	28,931 (56.8)	<0.001
Vital signs			
Mean arterial pressure (mmHg)	79.0 ± 10.9	81.2 ± 14.1	<0.001
Heart rate (beats/min)	83.9 ± 15.5	84.7 ± 16.4	<0.001
Respiratory rate (breaths/min)	19.2 ± 3.7	19.6 ± 5.0	<0.001
Oxygen saturation (%)	96.9 ± 2.0	96.9 ± 2.3	0.07
Temperature °C)	36.8 ± 0.5	36.8 ± 0.8	0.02
Systolic blood pressure (mmHg)	116.9 ± 15.8	120.9 ± 19.3	<0.001
Diastolic blood pressure (mmHg)	63.3 ± 11.3	63.5 ± 12.2	0.003
Laboratory values			
Bicarbonate (mEq/L)	21.6 ± 4.7	23.6 ± 5.2	<0.001
Glucose (mg/dL)	167.6 ± 99.6	155.4 ± 80.2	<0.001
Creatinine (mg/dL)	1.6 ± 1.7	1.6 ± 1.7	0.207
White blood cells (×10⁹/L)	14.4 ± 8.2	13.6 ± 10.3	<0.001
Lactate (mmol/L)	3.1 ± 2.6	2.8 ± 2.9	<0.001
Troponin (ng/mL)	0.3 ± 1.5	1.5 ± 6.9	<0.001
Platelet count (×10⁹/L)	183.6 ± 96.9	191.4 ± 94.6	<0.001
Hemoglobin (g/dL)	9.8 ± 2.3	10.5 ± 2.3	<0.001
pH	7.3 ± 0.1	7.0 ± 0.3	<0.001
Clinical scores			
APACHE II	21.1 ± 7.7	17.9 ± 6.5	<0.001
SOFA score	4.9 ± 3.4	3.7 ± 2.8	<0.001
Derived clinical features			
Low MAP, *n* (%)	31,286 (68.0)	33,955 (66.6)	<0.001
Elevated lactate, *n* (%)	17,417 (37.9)	5,873 (11.5)	<0.001
Tachycardia, *n* (%)	22,184 (48.2)	30,065 (59.0)	<0.001
Hypothermia, *n* (%)	7,871 (17.1)	9,709 (19.1)	<0.001
Hypoxemia, *n* (%)	9,708 (21.1)	25,154 (49.4)	<0.001
Cardiac Comorbidities			
Acute myocardial infarction, *n* (%)	7,171 (15.6)	4,708 (9.2)	<0.001
Heart failure, *n* (%)	15,997 (34.8)	5,744 (11.3)	<0.001
Coronary artery disease, *n* (%)	19,158 (41.6)	1,464 (2.9)	<0.001
Cardiac arrest, *n* (%)	6,728 (14.6)	2,180 (4.3)	<0.001
Arrhythmia, *n* (%)	14,664 (31.9)	6,737 (13.2)	<0.001
Treatment			
Vasopressor use, *n* (%)	11,793 (25.6)	8,187 (16.1)	<0.001
ICU admission details			
CCU admission, *n* (%)	8,454 (18.4)	11,330 (22.2)	<0.001
CVICU admission, *n* (%)	13,398 (29.1)	15,976 (31.4)	<0.001
Emergency admission, *n* (%)	19,021 (41.3)	49,361 (96.9)	<0.001
ICU length of stay (hours)	108.7 ± 145.8	93.4 ± 128.9	<0.001
Primary outcome			
Hemodynamic deterioration, *n* (%)	20,247 (44.0)	7,894 (15.5)	<0.001

Comorbidities and Severity: There was a marked difference in the disease profile between the two datasets. The MIMIC-IV cohort represented a significantly sicker population with a higher burden of cardiovascular comorbidities, including heart failure (34.8% vs. 11.3%), coronary artery disease (41.6% vs. 2.9%), and arrhythmias (31.9% vs. 13.2%). Disease severity scores were also higher in MIMIC-IV (APACHE II: 21.1 ± 7.7 vs. 17.9 ± 6.5; SOFA: 4.9 ± 3.4 vs. 3.7 ± 2.8), indicating a more critically ill population. Notably, emergency admissions were far more common in the eICU-CRD cohort (96.9%) compared to MIMIC-IV (41.3%).

Primary Outcome: Consistent with the higher severity scores and comorbidity burden, the incidence of the primary outcome—hemodynamic deterioration—was substantially higher in the MIMIC-IV cohort (44.0%, *n* = 20,247) compared to the eICU-CRD cohort (15.5%, *n* = 7,894; *P* < 0.001). These significant disparities in baseline characteristics and outcome prevalence between the two datasets underscore the necessity and value of our bidirectional validation strategy to ensure model robustness.

### Baseline model performance and optimal model selection

**Figure 2:**
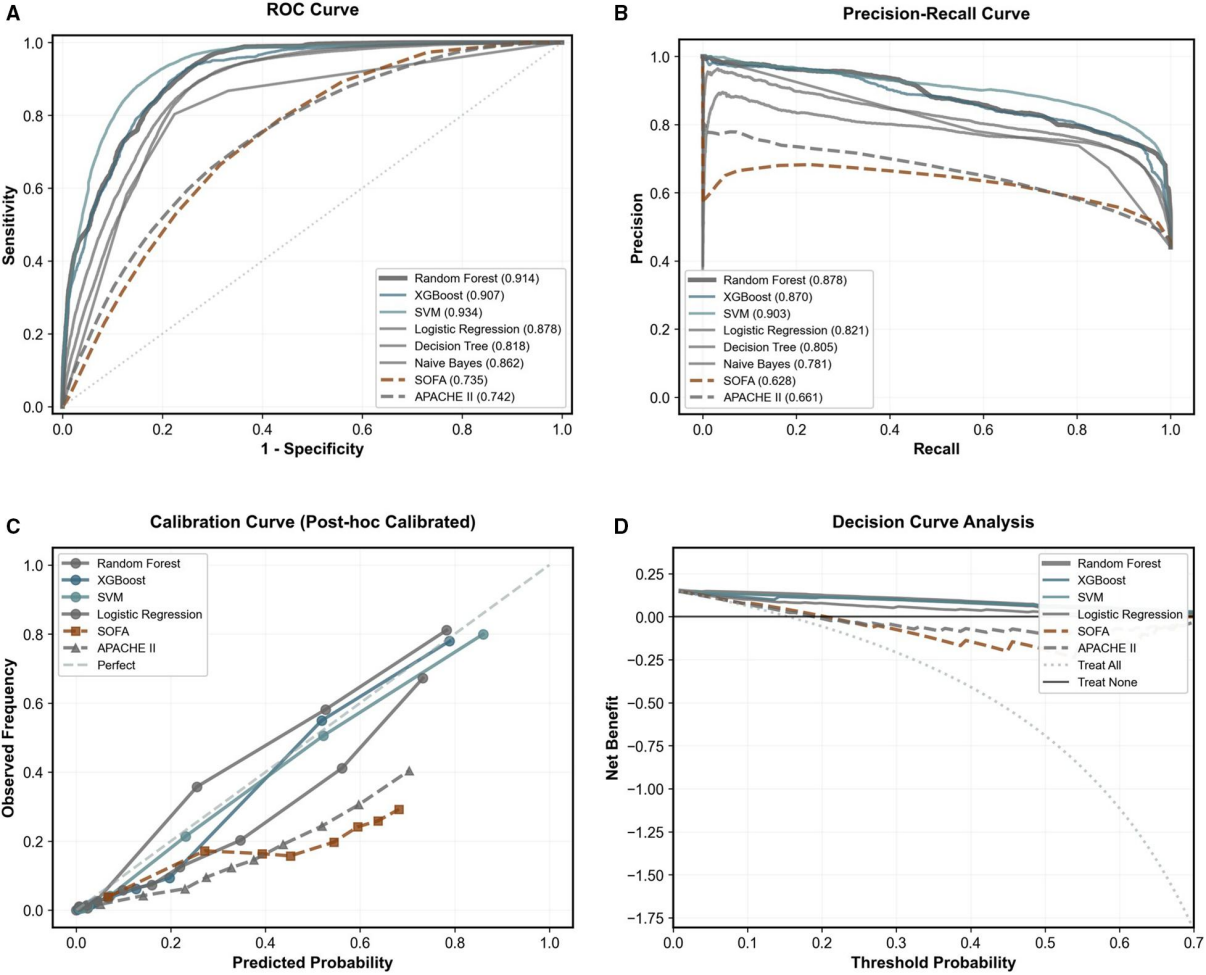
Performance evaluation of machine learning models compared with traditional clinical scoring systems in the external validation cohort. **(A)** ROC curves and **(B)** Precision-Recall curves showing the discrimination performance of six machine learning models vs. SOFA and APACHE II scores. SVM and Random Forest exhibited the leading performance metrics. **(C)** Calibration plots (post-hoc calibrated) displaying the agreement between predicted and observed risks. **(D)** Decision curve analysis demonstrating the clinical net benefit of the models. The machine learning models consistently showed higher net benefit than traditional clinical scores across relevant probability thresholds.

The candidate machine learning models in Phase 1 demonstrated robust performance in external validation (Figure 2). ROC curve analysis showed that SVM achieved the highest AUROC of 0.934, closely followed by Random Forest at 0.914 and XGBoost at 0.907. In precision-recall curve analysis, SVM showed the best AUPRC of 0.903, while Random Forest maintained a competitive AUPRC of 0.878.

Calibration plot analysis revealed differences in model prediction reliability. Most machine learning models showed good calibration across the probability intervals, significantly outperforming the clinical scores. Decision curve analysis indicated that within the wide threshold probability range, both SVM and Random Forest models provided substantial clinical net benefit.

Despite the marginally higher metrics of SVM, the Random Forest model was selected as the optimal model for final deployment. This decision was driven by its superior clinical interpretability and robustness against overfitting. Unlike SVM, Random Forest provides intrinsic feature importance rankings, offering transparency crucial for clinical decision-making. Furthermore, as an ensemble method, it demonstrated greater stability in handling the heterogeneity of clinical data compared to the kernel-based SVM. After systematic hyperparameter optimization, the finalized Random Forest model achieved an AUROC of 0.914 on the test set, balancing high predictive accuracy with necessary clinical explainability.

### Bidirectional modeling and cross-validation results

**Figure 3:**
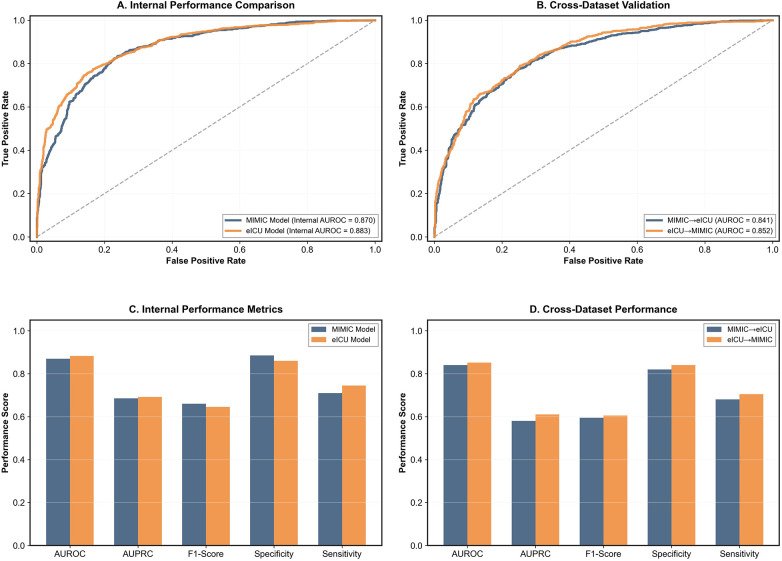
Bidirectional performance evaluation and cross-dataset validation of the random forest models. **(A)** Receiver Operating Characteristic (ROC) curves comparing the internal validation performance of models trained on the MIMIC dataset (blue, AUROC = 0.870) and the eICU dataset (orange, AUROC = 0.883). **(B)** ROC curves for cross-dataset external validation. The blue line represents the MIMIC-trained model tested on the eICU dataset (AUROC = 0.841), and the orange line represents the eICU-trained model tested on the MIMIC dataset (AUROC = 0.852). **(C)** Comparison of internal performance metrics (AUROC, AUPRC, F1-Score, Specificity, and Sensitivity) for both models on their respective source datasets. **(D)** Comparison of cross-dataset performance metrics, illustrating the generalizability of the models when applied to the external dataset.

Based on the optimally selected Random Forest algorithm from Phase 1, bidirectional prediction models were constructed (Figure 3). Internal performance comparison showed that both MIMIC-trained and eICU-trained models achieved similar high performance levels on their respective datasets (AUROC of 0.870 and 0.883, respectively). Cross-dataset validation results demonstrated MIMIC → eICU performance of AUROC 0.841 and eICU → MIMIC of 0.852, showing excellent cross-database generalizability.

Internal performance metrics comparison showed that both models achieved similar high levels across AUROC, AUPRC, F1-score, specificity, and sensitivity. Cross-dataset performance analysis further confirmed model robustness, with performance decreases controlled within acceptable ranges (MIMIC model performance decrease 3.3%, eICU model performance decrease 3.5%). Bootstrap resampling analysis (*n* = 1,000) provided reliable confidence interval estimates for model performance. Both model distributions showed good stability and reproducibility, confirming reliable model performance.

### Clinical decision support and risk stratification

Threshold strategy comparison identified distinct operational points for clinical application: a Balanced strategy (Youden Index) at a threshold of *θ* = 0.32 and a High Specificity strategy at *θ* = 0.85. The Balanced strategy maintained high sensitivity (>90%) while preserving adequate specificity, whereas the High Specificity threshold maximized the true negative rate, offering flexible options for varying clinical resource allocation scenarios.

**Figure 4:**
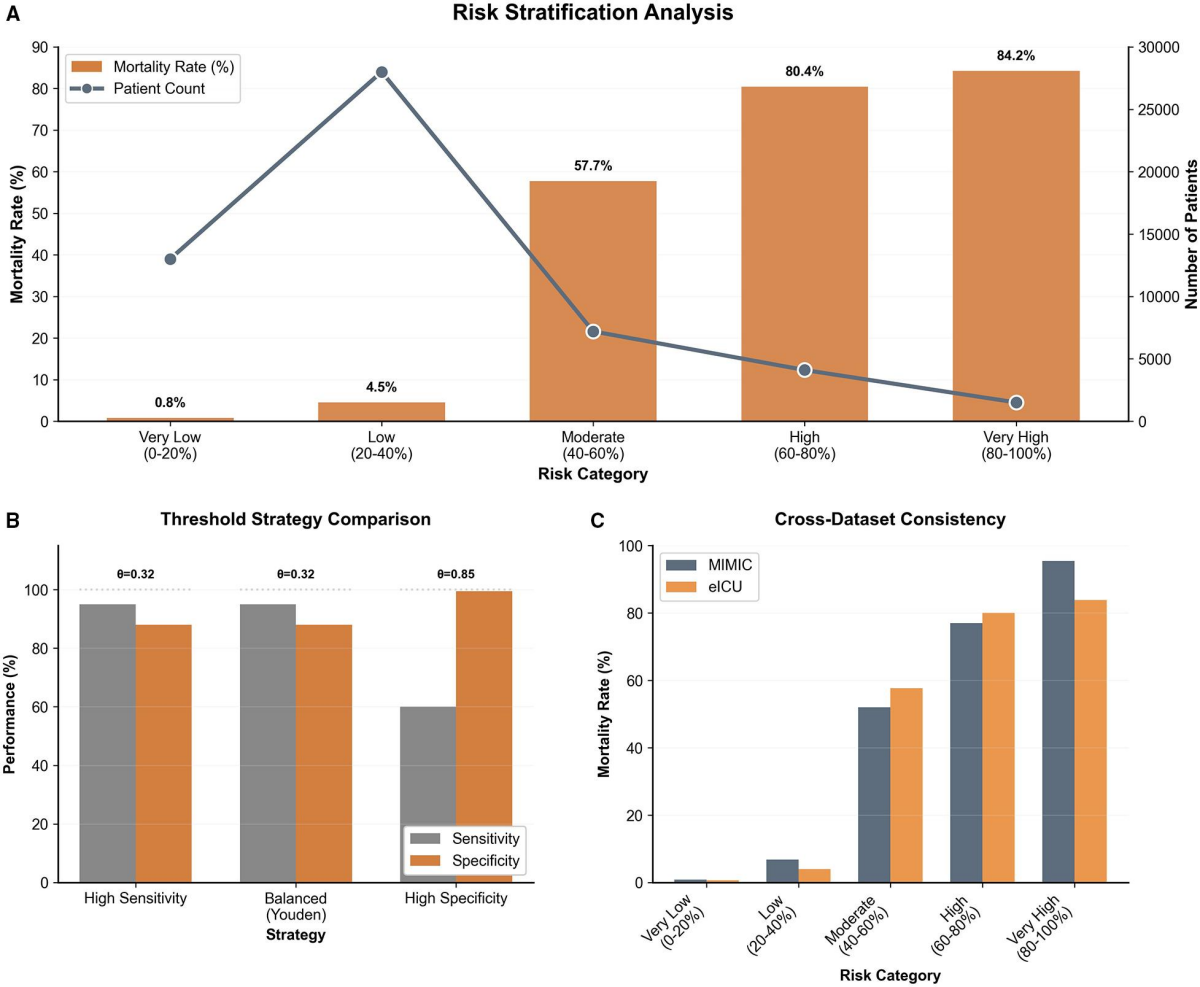
Risk stratification and clinical utility analysis **(A)** risk stratification analysis. Patients were stratified into five risk categories based on predicted probabilities (from Very Low 0%–20% to Very High 80%–100%). The orange bars illustrate a monotonic increase in actual mortality rates, rising sharply from 0.8% in the very low-risk group to 84.2% in the very high-risk group. The grey line (secondary *y*-axis) depicts the distribution of patient volume across groups, indicating that the majority of patients fall within the lower risk categories. **(B)** Threshold Strategy Comparison. This chart demonstrates the trade-offs in model performance under different decision thresholds ($\theta$). It compares Sensitivity (grey) and Specificity (orange) across three strategies: High Sensitivity strategy, Balanced strategy (optimal threshold *θ* = 0.32 derived from the Youden Index), and High Specificity strategy (*θ* = 0.85). **(C)** Cross-Dataset Consistency. A comparison of mortality rates across the five risk categories between the MIMIC and eICU cohorts. The results show consistent risk gradients across both datasets, validating the robustness and generalizability of the risk stratification system across different clinical populations.

The risk stratification analysis demonstrated excellent discriminatory power across five distinct categories based on predicted probability ranges (0%–20% to 80%–100%) (Figure 4). The mortality rates exhibited a strict monotonic increase: 0.8% in the Very Low risk group (0%–20%), rising to 4.5% (Low), 57.7% (Moderate), 80.4% (High), and peaking at 84.2% in the Very High risk group (80%–100%). Notably, the sharp elevation in mortality between the Low (4.5%) and Moderate (57.7%) groups highlights the model's effectiveness in distinguishing stable patients from those at escalating risk.

Decision Curve Analysis (DCA) further validated the clinical utility. As shown in the calibration and decision curves, the model's net benefit consistently exceeded both “treat all” and “treat none” strategies across a broad range of threshold probabilities. The Random Forest and XGBoost classifiers, in particular, demonstrated superior net benefit, confirming their practical value for informing clinical interventions.

Cross-dataset consistency analysis between the MIMIC and eICU cohorts confirmed the robustness of this stratification system. Both datasets showed comparable mortality trends across all risk categories. While the MIMIC dataset showed slightly higher mortality in the Very High risk category compared to eICU, the overall escalating trend remained stable, demonstrating the system's generalizability across different critical care populations.

### Model interpretability and feature analysis

**Figure 5:**
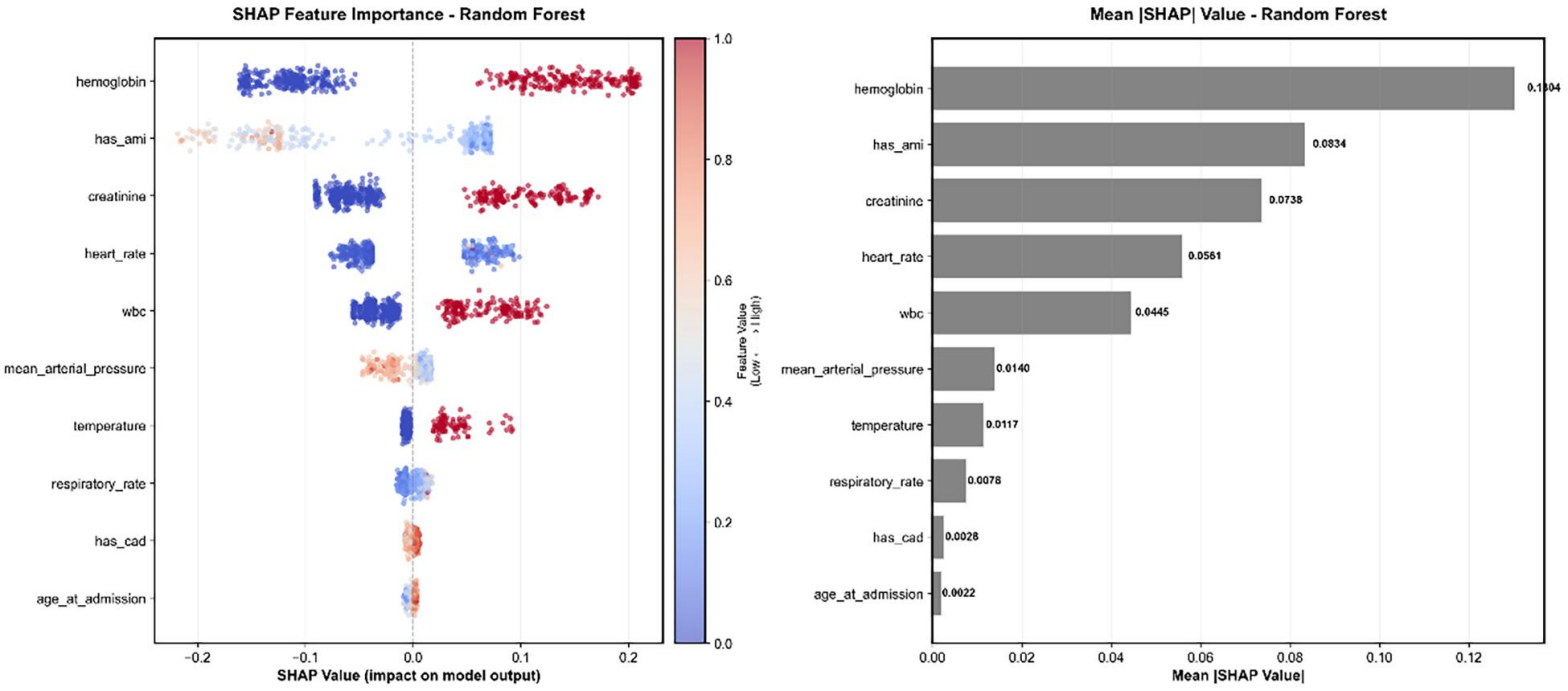
SHAP feature importance analysis for the random forest model. The left panel displays the SHAP summary beeswarm plot, illustrating the directional impact of feature values on model output. Each dot represents an individual sample; color indicates the feature value (red for high, blue for low), and the *x*-axis position represents the SHAP value (positive values indicate a contribution to higher risk prediction, negative values indicate lower risk). The right panel shows the feature importance ranking based on the mean absolute SHAP value, identifying hemoglobin, history of AMI (has_ami), and creatinine as the top three predictors.

SHAP analysis revealed the clinical logic of model decisions (49, 50) (Figure 5). Feature importance ranking showed that hemoglobin (0.1304), history of acute myocardial infarction (AMI) (0.0834), and creatinine (0.0738) were the most important predictors, reflecting the central role of hemodynamic parameters in risk prediction. Feature importance consistency analysis showed high correlation between different models (*r* = 0.980), demonstrating stable predictive logic.

Clinical feature category analysis showed that laboratory indicators (including hemoglobin, creatinine, and WBC) accounted for the largest proportion of feature importance, surpassing **vital signs** (heart rate, mean arterial pressure, temperature). This distribution suggests that metabolic and organ function markers play a more critical role than transient hemodynamic fluctuations in this specific model.

Detailed feature analysis revealed distinct relationships between key features and outcomes. Hemoglobin exhibited the highest feature importance, with higher values generally associated with increased model output in this cohort. Creatinine and White Blood Cell (WBC) count showed a clear positive correlation with risk, where elevated values (indicated by red points on the right side of the SHAP plot) corresponded to higher risk scores, reflecting the impact of renal dysfunction and inflammation/infection on critical illness. Interestingly, history of AMI displayed a negative contribution to the risk score (red points associated with negative SHAP values), potentially reflecting specific care protocols or selection biases within the ICU population. Heart rate maintained its relevance as a vital sign, with tachycardia positively correlating with higher risk.

### Comparison of model performance and statistical validation

Following the identification of key predictive features via SHAP analysis, we further validated the performance superiority of the Random Forest model against traditional clinical scores and other machine learning algorithms. As shown in Table 2, pairwise comparisons of the Area Under the Receiver Operating Characteristic Curves (AUROCs) were performed using the DeLong test.

**Table 2:** Delong test comparison of AUROCs between the random forest model and other predictive methods.

Comparison	AUROC (Random forest)	AUROC (Comparator)	Difference (95% CI)	Z-statistic	*P* value
vs. Traditional clinical scores					
Random Forest vs. SOFA	0.949	0.681	0.267 (0.262–0.273)	91.53	<0.001
Random Forest vs. APACHE II	0.949	0.747	0.202 (0.196–0.207)	71.1	<0.001
vs. Other machine learning models					
Random Forest vs. XGBoost	0.949	0.911	0.038 (0.036–0.040)	36.09	<0.001
Random Forest vs. Logistic Regression	0.949	0.872	0.077 (0.074–0.080)	45.05	<0.001

AUROC, area under the receiver operating characteristic curve; CI, confidence interval; SOFA, sequential organ failure assessment; APACHE II, acute physiology and chronic health evaluation II.

*P* values were calculated using the DeLong test for two correlated ROC curves.

The Random Forest model achieved the highest AUROC of 0.949. It demonstrated significant superiority over traditional clinical scoring systems, with an AUROC significantly higher than both the SOFA score (0.681; difference 0.267, *P* < 0.001) and the APACHE II score (0.747; difference 0.202, *P* < 0.001). Furthermore, when compared to other machine learning models, the Random Forest model showed a statistically significant performance improvement over XGBoost (0.911, *P* < 0.001) and Logistic Regression (0.872, *P* < 0.001). These results confirm the robustness and significant advantage of the Random Forest model in predictive accuracy.

### Performance across clinical subgroups

We conducted a comprehensive subgroup analysis on the external validation set to assess whether the Random Forest model's performance varied across different patient profiles. As shown in Table 3, the model remained robust across all subgroups.

**Table 3:** Fairness analysis.

Subgroup analysis	*N*	Events (%)	AUROC	95% CI	Z-statistic	*P* value
Demographics						
Sex						
Female	22,018	14.5	0.841	0.835–0.847	—	—
Male	28,931	16.3	0.848	0.844–0.853	2	0.045
Age group						
<65 years	23,540	11.2	0.849	0.843–0.855	—	—
≥65 years	27,409	19.2	0.84	0.835–0.844	−2.41	0.016
Cardiac comorbidity						
History of cardiac arrest						
No	48,769	14.9	0.846	0.843–0.850	—	—
Yes	2,180	27.8	0.834	0.820–0.848	−1.7	0.089
Disease severity						
SOFA score						
<6	38,143	11.9	0.834	0.829–0.839	—	—
≥6	12,806	26.2	0.866	0.862–0.871	9.03	<0.001
APACHE II score						
<15	16,568	4	0.817	0.802–0.831	—	—
≥15	34,381	21.1	0.843	0.839–0.846	4.54	<0.001

AUROC, area under the receiver operating characteristic curve; CI, confidence interval; SOFA, sequential organ failure assessment; APACHE II, acute physiology and chronic health evaluation II.

All analyses were performed on the eICU-CRD external validation cohort (*n* = 50,949). *P* values were calculated using the *Z*-test for comparing correlated AUROC values between subgroups. References groups (indicated by “—”) represent the baseline comparator within each category.

While statistical differences were noted in demographic comparisons—with slightly higher AUROCs in males (0.848 vs. 0.841, *P* = 0.045) and younger patients (0.849 vs. 0.840, *P* = 0.016)—the absolute differences were minimal, indicating stable performance across age and sex.

Crucially, the model demonstrated stronger predictive power in critically ill patients. Stratification by disease severity revealed that the model achieved its highest performance in patients with a SOFA score ≥6 (AUROC 0.866, 95% CI 0.862–0.871), significantly outperforming the low-SOFA subgroup (*P* < 0.001). This pattern was corroborated by the APACHE II stratification, where patients with scores ≥15 showed significantly better discrimination (AUROC 0.843) than those with scores <15 (*P* < 0.001). These findings suggest that the model is particularly effective at identifying risk in the most severely ill patient populations.

## Discussion

This study successfully developed and validated a machine learning-based early warning system for hemodynamic deterioration in cardiovascular ICU patients, demonstrating robust performance through bidirectional cross-validation across two large critical care databases. The achieved AUROC of 0.914 and excellent cross-dataset generalizability (performance decrease <4%) represent significant advances in cardiovascular critical care prediction, particularly given the rigorous external validation methodology employed.

### Clinical significance and comparison with existing literature

Our results align with and extend recent advances in ML-based clinical deterioration prediction (22–24). The achieved performance metrics are comparable to or exceed those reported in recent cardiovascular ICU prediction studies (32, 33). The bidirectional validation strategy employed in this study addresses a critical limitation identified in recent systematic reviews, where only 5%–7.1% of published ML studies in ICU settings performed external validation (37, 38). By demonstrating consistent feature importance and stable performance across datasets (MIMIC-IV and eICU), we provide compelling evidence for the model's robust generalizability across diverse healthcare environments.

The multi-level risk stratification system developed in this study represents a novel contribution to cardiovascular ICU management. Unlike binary prediction models commonly reported in the literature, our five-level stratification system enables precise resource allocation and individualized treatment intensification. This approach addresses the practical clinical need for nuanced risk assessment rather than simple high/low risk categorization.

The early warning value of our system is particularly noteworthy. The system can provide warnings 2–6 h before hemodynamic deterioration occurs, securing valuable time windows for clinical intervention. Using a single predictive model, we can adapt to different clinical needs by adjusting decision thresholds: the high-sensitivity screening model (89.4% sensitivity) ensures timely identification of high-risk patients, while the high-specificity confirmation model (80.6% specificity) reduces unnecessary clinical interventions. SHAP analysis-provided individualized explanations further enhance the model's clinical interpretability, allowing physicians to understand specific clinical factors driving predictions (50).

## Limitations

As a retrospective analysis, this study has several important limitations that must be acknowledged. First, the retrospective design limits our ability to control for variations in clinical practices, data collection protocols, and treatment decisions across different time periods and institutions. The MIMIC-IV database spans 2008–2019 while eICU covers 2014–2015, potentially introducing temporal biases related to evolving medical practices and technologies.

Second, despite rigorous data quality control measures, retrospective studies are susceptible to coding errors, missing data patterns, and selection biases that may not be fully captured through computational methods. While our multiple imputation strategies were based on established clinical principles, they may not fully represent the true clinical complexity and individual patient variability encountered in real-world scenarios.

Third, the study's reliance on structured electronic health record data limits the incorporation of important clinical nuances, such as nursing assessments, physician clinical judgment, and patient-reported symptoms that often contribute to hemodynamic status evaluation. The model was developed based on data from North American healthcare systems, and its applicability in other healthcare systems, different populations, or resource-limited environments requires further validation.

The retrospective nature of this study necessitates prospective validation to establish clinical utility and real-world effectiveness. We are currently developing collaborations with multiple healthcare institutions to conduct prospective clinical trials that will evaluate the system's impact on patient outcomes, clinical workflow integration, and healthcare resource utilization.

Collaboration with partner hospitals will enable multi-center validation across diverse patient populations, healthcare systems, and clinical practices.

### Methodological innovations and clinical translation

The bidirectional validation approach implemented in this study establishes a new standard for ML model evaluation in critical care settings (41–44). This methodology provides more robust assessment of model generalizability than traditional single-database validation, identifying both consistent predictive patterns and dataset-specific variations that may limit clinical applicability. This methodology effectively identifies both consistent predictive patterns and dataset-specific variations, ensuring the model's reliability across diverse populations.

Successful clinical implementation of ML-based early warning systems requires careful consideration of several factors beyond predictive accuracy (50). The interpretability analysis provided through SHAP methodology represents an important step toward clinical acceptance by providing transparent, clinically meaningful explanations for model predictions (45–47, 49, 50). However, real-world deployment faces additional challenges including system integration with existing electronic health records, alert delivery mechanisms, and clinical response protocols.

## Conclusions

This study demonstrates the feasibility and clinical potential of ML-based early warning systems for hemodynamic deterioration in cardiovascular ICU patients. The bidirectional validation approach provides robust evidence for model generalizability, while the multi-level risk stratification system offers practical clinical utility. The main innovations of this warning system include: (1) pioneering bidirectional cross-validation design ensuring cross-dataset generalization ability; (2) dual-target variable system meeting different clinical application needs; (3) rigorous feature auditing ensuring clinical operability; (4) SHAP interpretability analysis enhancing clinical acceptance. Furthermore, DeLong tests confirmed the statistical superiority of our models over traditional scores, while fairness analysis demonstrated consistent performance across diverse patient subgroups, mitigating potential algorithmic bias.

Research results indicate that this warning system has the potential to improve early identification rates of hemodynamic deterioration in clinical practice, enhance patient outcomes, and optimize healthcare resource utilization efficiency. However, the retrospective nature of the study necessitates prospective validation and real-world implementation studies to establish clinical effectiveness. The planned hospital collaborations and prospective trials represent critical next steps toward translating these research findings into improved patient care and clinical outcomes.

## Data Availability

The original contributions presented in the study are included in the article/Supplementary Material, further inquiries can be directed to the corresponding author.
